# Ultra-strong polymeric hollow fiber membranes for saline dewatering and desalination

**DOI:** 10.1038/s41467-021-22684-1

**Published:** 2021-04-20

**Authors:** Can Zeng Liang, Mohammad Askari, Looh Tchuin (Simon) Choong, Tai-Shung Chung

**Affiliations:** 1 Department of Chemical & Biomolecular Engineering, National University of Singapore, Singapore, Singapore; 2 Gradiant International Holdings Pte. Ltd., 1 Cleantech Loop #03-04/05/06, Singapore, Singapore; 3 Graduate Institute of Applied Science and Technology, National Taiwan University of Science and Technology, Taipei, Taiwan

**Keywords:** Chemical engineering, Composites, Water resources

## Abstract

Osmotically assisted reverse osmosis (OARO) has become an emerging membrane technology to tackle the limitations of a reverse osmosis (RO) process for water desalination. A strong membrane that can withstand a high hydraulic pressure is crucial for the OARO process. Here, we develop ultra-strong polymeric thin film composite (TFC) hollow fiber membranes with exceptionally high hydraulic burst pressures of up to 110 bar, while maintaining high pure water permeance of around 3 litre/(m^2^ h bar) and a NaCl rejection of about 98%. The ultra-strong TFC hollow fiber membranes are achieved mainly by tuning the concentration of the host polymer in spinning dopes and engineering the fiber dimension and morphology. The optimal TFC membranes display promising water permeance under the OR and OARO operation modes. This work may shed new light on the fabrication of ultra-strong TFC hollow fiber membranes for water treatments and desalination.

## Abstract (web summary)

Osmotically assisted reverse osmosis can overcome limitations of the reverse osmosis process but a strong membrane which can withstand a high hydraulic pressure is crucial. Here, the authors develop strong polymer thin film composite hollow fiber membranes with exceptionally high hydraulic burst pressures of up to 110 bar, while maintaining high water permeance and salt rejection.

## Introduction

Currently, reverse osmosis (RO) desalination is the most energy-efficient process for producing clean drinking water from seawater and/or saline water when compared with the conventional thermal processes^1–3^. The maximum water recovery of the conventional RO process is about 35–50%^4–6^, mainly due to limitations stemmed from a maximum salinity (e.g., >70 g/L) of the feed stream and practical considerations such as mechanical strength of membranes, economic and environmental concerns^3,7,8^. With continuing water recovery, the saline feed becomes more concentrated. Thus, the RO process must consume extra energy to overcome the osmotic pressure exerted by the concentrated saline water^2,9^. Additionally, the treatment of the concentrated RO effluent stream is not cheap. For instance, 5–33% of the total cost of the RO desalination process is spent on the disposal of the RO effluent^3,7^.

In order to maximize the RO potential and increase its water recovery, the desalination of the highly saline water has recently received increasing attention from academia and industries^3,6^. One of the promising technologies to realize high water recovery (e.g., >50%) is the membrane-based osmotically assisted reverse osmosis (OARO) process^3,10–14^. In the OARO process, water is transported across the semi-permeable membrane driven by the hydraulic pressure that overcomes the transmembrane osmotic pressure difference^3,14^. Briefly, a saline stream with a lower or equal salinity is employed in the permeate side as a sweep stream of the OARO process to reduce the difference of osmotic pressure across the membrane, thereby the water transport becomes possible even when the osmotic pressure of the feed is larger than the external applied hydraulic pressure^3,14^. Therefore, OARO makes a high water recovery of >70% via a membrane-based energy-efficient process possible^3,11,13,14^. Nevertheless, the OARO process is also limited by the burst pressure of the membrane as the water recovery becomes higher^3,11,13,14^. Thus, the development of strong and efficient membranes is a key in meeting the requirements of the OARO processes.

The conventional flat sheet or spiral-wound RO membranes usually are able to withstand a maximum pressure of about 80 bar^3,15^. Although the operating pressure of spiral-wound RO membrane modules could be further increased to 120 bar by employing a special membrane and module design, the effective membrane area inside the special module decreases by about 6 times as compared to a typical similar-sized RO module (e.g., ~ 6 vs. ~37 m^2^)^3,15^. Besides, the water transport resistance increases significantly when the special spiral-wound module is operated under high pressures due to the compaction of both the spacer and support layer^16–18^. By contrast, the hollow fiber membrane is self-supported and has a higher surface-to-volume ratio than the flat-sheet membrane. Besides, the hollow fiber membrane is easier to scale up and simpler in module fabrication than the flat-sheet one^19,20^.

Currently, the leading hollow fiber membranes suitable for the OARO process are produced by Toyobo (Japan) whose cellulose triacetate (CTA) hollow fibers have been used extensively in various desalination industries. Toyobo’s pressure retarded osmosis (PRO) and RO hollow fiber membranes can withstand the maximum pressures of up 29 bar and 69 bar under the out-to-in mode (i.e., from the shell side to the lumen), respectively^21^. However, the pressure drops of its hollow fiber modules are considerably high because its hollow fiber membranes are outer selective (i.e., the selective layer is on the shell side) and small in dimensions (i.e., inner diameter/outer diameter = ~80/180 µm)^13,21–23^. For example, Togo et al. used Toyobo’s PRO hollow fiber membranes for OARO, in which a low pressure of 8–18 bar was applied at the shell side. They reported that the pressure drop along the lumen side could be up to about 30–60% of the operating pressures depending on the flow rate^13^.

To improve the mechanical strength and the crush or burst pressure of hollow fiber membranes, numerous strategies were followed (1) adding woven^24^, carbon nanotubes^25^, and inorganic salts^26,27^; (2) adopting dual-layer extrusion technology^28,29^; (3) employing the braid reinforced elements^30–32^. However, to the best of our knowledge, the aforementioned hollow fiber membranes are not ideal or suitable for OARO because they are mechanically weak (e.g., having a burst pressure less than 35 bar). Recently, Jang et al. developed a single-layer asymmetric hollow fiber membrane (made from Torlon) that could withstand a crushing pressure (out-to-in pressure) of 95 bar but the permeance for organic solvent nanofiltration is low^33^. Therefore, the fabrication of ultra-strong hollow fiber membranes with reasonable permeance is challenging but highly in demand for OARO processes.

The objective of this work is to understand the fundamental science and engineering aspects onto the design of ultra-strong inner-selective thin-film composite (TFC) hollow fiber membranes. We aim to produce TFC hollow fibers with a high burst pressure of ~100 bar so that they are applicable for desalination and dewatering from saline water via both RO and OARO operation modes. Interfacial polymerization was chosen to synthesize the selective layer because the resultant polyamide layer would have a high NaCl rejection suitable for desalination^34,35^. Meanwhile, the inner-selective configuration was selected because this configuration would be easy for subsequent scale up and production^36,37^. To achieve the goal, two strategies were deployed: (1) increasing the polymer concentration of the spinning dopes, and (2) tuning the dimension of hollow fibers by adjusting their inner diameter and wall thickness. Various characterization techniques were also conducted to elucidate the evolution of membrane morphology and performance as a function of fabrication conditions. The as-developed ultra-strong TFC hollow fiber membranes may not only be suitable for OARO applications but also promising to enhance the energy efficiency and increase the water recovery of conventional RO desalination plants. In addition, the strong TFC hollow fibers potentially can be employed in a RO-OARO integrated high-pressure desalination process to achieve a higher water recovery in a more economic and environment-friendly way.

## Results

### Characteristics of PES hollow fiber membrane substrates

For a TFC hollow fiber membrane, the mechanical strength is dominated or controlled by the hollow fiber membrane substrate because the mechanical strength of the ultrathin selective layer of <1 µm is low. Therefore, the membrane substrates are firstly fabricated and optimized by tuning the PES polymer concentration, their wall thickness, and morphologies. Table 1 summarizes their physical and mechanical properties. As the ratio of the dope to bore fluid flow rate (hereafter, designated as the dope/bore fluid ratio) increases from 1 to 10, the outer diameters (OD) of hollow fibers are maintained at about 1000 µm, while the inner diameters (ID) decrease from about 640 to 300 µm. As a result, the wall thicknesses of the hollow fibers increase from ~200 to 360 µm. Consistent with the increases in PES concentration and wall thickness, the burst pressure of the hollow fiber substrates also increases from 17 to 83 bar. The burst pressure is the maximum pressure that a hollow fiber membrane can withstand under an in-to-out testing mode. Once the burst pressure is reached, the gauge pressure of the testing device suddenly decreases as a result of membrane failure.

**Table 1:** Physical and mechanical properties of the as-spun hollow fiber membrane substrates.

PES membrane substrate			Outer diameter (µm)	Inner diameter (µm)	Wall thickness (µm)	Bulk porosity (%)	Max. tensile strain (%)	Max. tensile stress (MPa)	Young’s modulus (MPa)	Burst pressure (bar)
PES (wt%)	Ratio of dope/bore fluid	Code name								
22	1	P22-A	914	543	186	75.1	39	4.88	186	17
	2	P22-B	900	443	229	75.2	66	5.39	184	27
	4	P22-C	984	379	302	73.7	76	5.50	180	43
	10	P22-D	1060	295	383	74.3	72	5.25	168	65
26	1	P26-A	1053	599	227	70.2	47	6.28	234	28
	2	P26-B	997	502	248	68.9	61	7.22	250	37
	4	P26-C	984	390	297	68.1	69	6.93	243	48
	10	P26-D	1019	290	365	68.8	77	7.35	283	79
30	1	P30-A	1131	643	244	69.7	50	7.36	259	30
	2	P30-B	1066	533	266	64.2	51	9.31	328	44
	4	P30-C	1045	443	301	63.9	59	8.77	301	59
	10	P30-D	1033	322	355	65.8	52	7.95	295	83

**Fig. 1:**
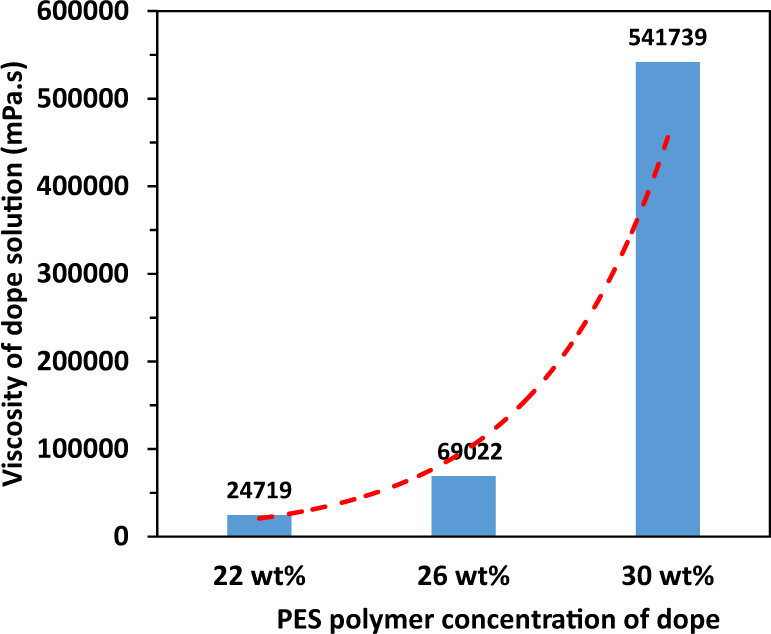
Bulk viscosities of the polymer dopes (composition: PES/NMP/H_2_O/CaCl_2_) with different PES concentrations. The dash line (red) is an exponential trend line for the sake of visualizing the increase of the viscosity with the PES concentration.

Overall, the bulk porosity of all substrates varies from about 65 to 75%. Generally, the bulk porosity declines with an increase in PES polymer concentration. Interestingly, even though the dope/bore fluid ratio increases from 1 to 10, the resultant substrates spun from the same dope solution have almost similar bulk porosities. Besides, there is a down-and-up trend for the bulk porosities as a function of the dope/bore fluid ratio. The bulk porosity decreases slightly as the ratio increases from 1 to 4 but increases slightly when the ratio reaches 10. This down-and-up trend possibly arises from the combination of 3 factors. (1) Since the dope viscosity increases exponentially with the PES concentration as shown in Fig. 1, it significantly alters the phase inversion process, die swelling phenomenon, and non-solvent intrusion mechanism^19,38–44^. (2) Because an increase in dope/bore fluid ratio results in a thicker fiber wall, this would cause the delay of solvent exchange between the solvent (NMP) and non-solvent (water) that may lead to a less porous structure or a lower bulk porosity^19,39,40^. (3) As the dope/bore fluid ratio increases to 10, the flow rate of bore fluid becomes very small (0.2 ml/min). This leads to a rapid change of bore fluid composition from pure water to a solvent (NMP) enriched mixture because of the solvent exchange between the bore fluid and the dope solution. The solvent enriched bore fluid would lead to a different phase inversion path and result in a more porous inner structure and thus a slightly higher bulk porosity.

**Fig. 2:**
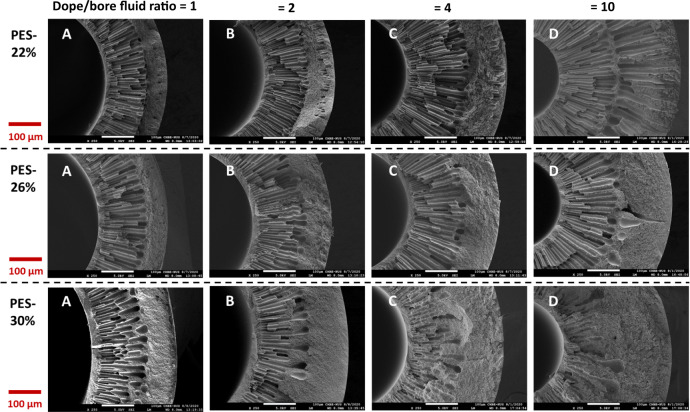
A, B, C, D represent the simplified code names of PES hollow fiber membranes with specific ratio of dope to bore fluid flow rate as shown on top of the figure, and in Tables 1 and 2. FESEM morphologies of selected cross-sections of the as-spun PES hollow fiber membrane substrates.

The above rationales are evidenced by the FESEM morphologies of the hollow fiber substrates. Supplementary Figure 1 shows their overall cross-section morphologies, while Fig. 2 displays their enlarged morphologies as a function of PES concentration in spinning dopes and dope/bore fluid ratio during spinning. Generally, the finger-like macrovoids in the cross-section decrease whilst the sponge-like microstructure increases with an increase in dope/bore fluid ratio and PES concentration. A macrovoid structure tends to appear at the inner region of the hollow fiber substrate possibly due to the non-solvent intrusion from the lumen side and the effect of die swelling^38,39,42,43^ while the sponge-like structure is formed at the outer region owing to the delayed demixing. The die swell phenomenon is a relaxation phenomenon when the shear-oriented polymer chains return to their random coil state after exiting from the annular channel of a spinneret. The relaxation rapidly expands the polymer chains moving inward to the lumen side as well as outward as referred to the die swell^42,43^.

**Fig. 3:**
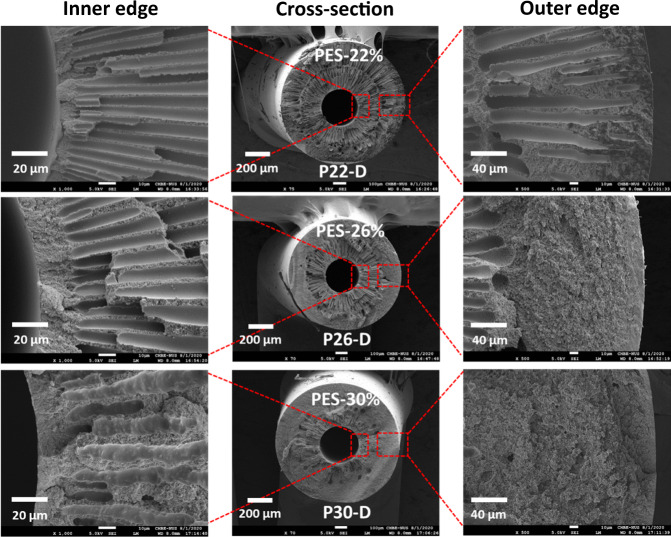
Highlighted FESEM morphologies of representative PES hollow fiber substrates. The hollow fibers were spun at the dope to bore fluid ratio of 10.

Figure 3 compares the FESEM morphologies of PES hollow fiber substrates spun from different PES concentrations but with the same dope/bore fluid ratio of 10. Interestingly, the hollow fiber (P22-D) spun from a dope containing a low PES concentration of 22 wt% has a dual-layer structure of finger-like macrovoids, while the others (P26-D and P30-D) possess only one layer of finger-like macrovoids. This phenomenon may result from the combined effects of the aforementioned factors. In other words, the nascent hollow fiber substrate spun from a dope with a low viscosity may experience non-solvent intrusion from both the internal and external coagulants, thus it has a dual-layer structure of finger-like macrovoids^39^. Once the dope viscosity is increased with the addition of PES, the nascent hollow fiber substrate can resist the non-solvent intrusion from the external coagulant because it has been relaxed from die swell in the air gap region and oriented by gravity and spinning-line stresses^38,39^. On the other hand, the inner region still has finger-like macrovoids because (1) the bore fluid and the dope meet immediately after extrusion from the spinneret and (2) the effect of inward die swelling may cause the non-solvent intrusion from the bore fluid.

In terms of mechanical properties and morphologies, Table 1 shows that the hollow fiber substrates with a dual-layer structure of finger-like macrovoids tend to have weaker mechanical properties (e.g., lower Young’s modulus and the maximal tensile stress) than the ones with a single layer of macrovoids. This is because finger-like macrovoids are the weak points of hollow fibers^19,38^. However, the dual macrovoid structure is favorable in reducing the internal concentration polarization (ICP), as discussed later.

**Fig. 4:**
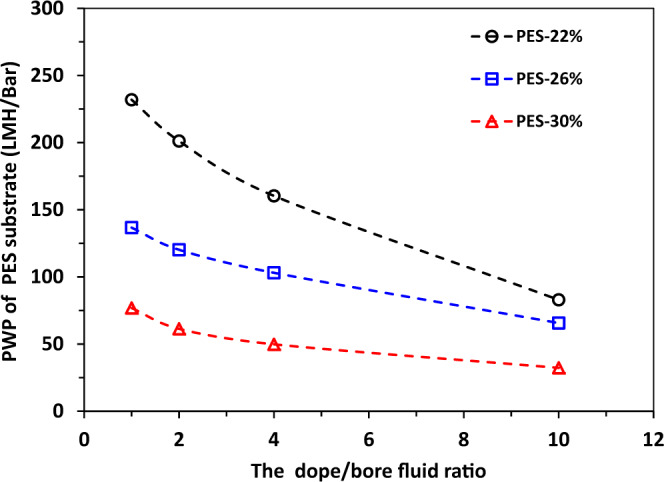
Pure water permeance (PWP) of the PES hollow fiber membrane substrates. The PWP measurement was conducted at a transmembrane pressure of 1 bar.

Supplementary Figure 2 shows the inner and outer surface morphologies of the substrates as a function of PES concentration spun at the dope/bore fluid ratio of 10. Both the inner and outer surfaces have relatively dense skin because water is used as the bore fluid and external coagulant. Figure 4 describes the pure water permeance (PWP) of these hollow fiber substrates as a function of the dope to bore fluid ratio. PWP varies in the range of approximately 230 to 30 litre/(m^2^ h bar) or LMH/bar, and decreases with the rise of the dope/bore fluid ratio. In addition, the substrate spun from a higher PES concentration has a lower PWP. The decline of PWP is because a higher dope/bore fluid ratio leads to a thicker membrane wall and a higher PES concentration results in a denser membrane structure. Both effects increase the mass transport resistance and lower the water permeance across the membranes.

### Characteristics of PES-TFC hollow fiber membranes

By performing the interfacial polymerization at the lumen side of the hollow fiber substrates, inner selective TFC hollow fiber membranes were fabricated. Table 2 depicts their characteristics including pure water permeance (A), NaCl rejection (R), salt permeability (B) and burst pressures. Since these PES-TFC membranes have different mechanical properties, they were tested under different conditions. For PWP and salt rejection tests, the PT22-A TFC membrane was conditioned and tested at 10 bar; PT26-A and PT30-A were conditioned and tested at 20 bar; all other TFC membranes were conditioned at 30 bar and tested at 20 bar. Most PWP values of TFC membranes are about 3 LMH/bar while their salt rejections are around 98%. Interestingly, although PWP of the hollow fiber substrates declines with an increase in PES concentration and dope/bore fluid ratio (Fig. 4), the PWP values of TFC membranes do not show a similar trend. This arises from the fact that the major transport resistance across the TFC membranes comes from the selective polyamide layer formed by the interfacial polymerization.

**Table 2:** Characteristics of the PES-TFC hollow fiber membranes^a^.

PES-TFC Membrane			PWP: A (LMH/bar)	Salt rejection: R (%)	Salt permeability: B (LMH)	Burst pressure (bar)
PES (wt%)	Ratio of dope/bore fluid	Code name				
22	1	PT22-A	3.13	98.5	0.40	22
	2	PT22-B	3.54	98.3	1.15	31
	4	PT22-C	3.00	97.3	1.52	52
	10	PT22-D	3.43	97.1	1.88	73
26	1	PT26-A	2.80	98.7	0.65	31
	2	PT26-B	2.94	98.1	1.04	47
	4	PT26-C	3.96	98.6	1.01	68
	10	PT26-D	3.64	98.3	1.15	104
30	1	PT30-A	1.81	98.2	0.61	35
	2	PT30-B	3.12	97.4	1.54	50
	4	PT30-C	2.98	98.4	0.88	77
	10	PT30-D	2.81	98.1	1.01	110

^a^For PWP and salt rejection tests, PT22-A TFC membrane was conditioned and tested at 10 bar; PT26-A and PT30-A were conditioned and tested at 20 bar; other TFC membranes were conditioned at 30 bar and tested at 20 bar.

### Polyamide layer

**Fig. 5:**
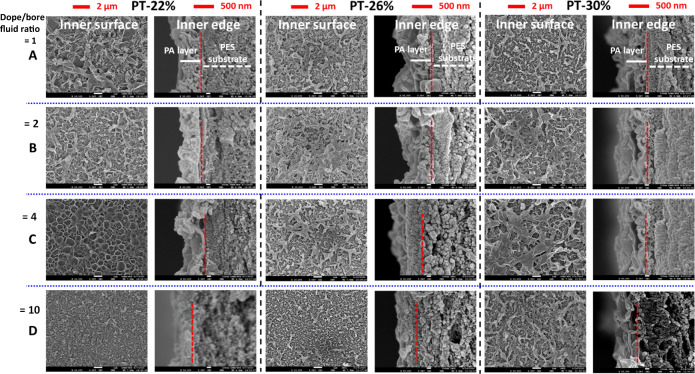
FESEM morphologies of the inner surface and inner edge of the cross-section of PES-TFC hollow fiber membranes. Polyamide (PA) layer was denoted as PA layer.

Figure 5 illustrates the FESEM images of the inner surfaces and inner edges of the PES-TFC membranes as a function of PES concentration and dope/bore fluid ratio. Generally, all polyamide selective layers possess a typical ridge-and-valley morphology due to the rapid interfacial polymerization reaction between MPD and TMC monomers^27,34–36^. The apparent thickness of the polyamide layers ranges from about 300 to 400 nm. Interestingly, the TFC membrane spun from a higher dope/bore fluid ratio tends to have a thinner polyamide layer. The decline in the polyamide thickness might be ascribed to the combined effects of capillary pressure and convective flow. Since the MPD solution is firstly circulated in the lumen side and absorbed near the inner surface region for the subsequent interfacial polymerization, the capillary pressure induced by the pores near the region and finger-like macrovoids would pull (suck) the MPD solution into the bulk membrane. According to the Young–Laplace equation, the capillary pressure is reciprocally proportional to the radius of a capillary^45,46^. A higher PES concentration would result in hollow fiber substrates with a smaller pore radius, while a higher dope/bore fluid ratio would produce substrates with a thicker fiber wall. The former would pull the MPD solution into the bulk substrate more effectively, whereas the latter would significantly retard the diffusion of MPD molecules back to the lumen surface to react with TMC. Thus, it leads to form a thinner TFC layer with an increase in PES concentration and dope/bore fluid ratio.

In addition, since the flow rate of the TMC solution is fixed during the IP process and the hollow fiber substrate spun from a higher dope/bore fluid ratio has a smaller inner diameter, this leads to a higher velocity of the TMC solution flowing through the smaller lumen (or smaller inner diameter) of the hollow fiber substrate. The fast-flowing TMC solution may lead to a shorter reaction time and constrains the growth of the polyamide layer on top of the inner surface. Besides, a fast TMC solution flowing through the lumen may result in interfacial polymerization not only on top of the inner surface but also within the pores near the inner-surface region. Thus, an apparent thinner polyamide layer is formed on top of the inner surface of the substrate.

### Burst pressure

As shown in Table 2, the burst pressures of the PES-TFC membranes range from 22 to 110 bar. Consistent with the burst pressures of their PES substrates as tabulated in Table 1, the PES-TFC membranes spun from the same PES concentration possess a higher burst pressure if the dope/bore fluid ratio is increased (e.g., due to a thicker wall). For PES-TFC membranes with a similar wall thickness, the one fabricated from a higher PES concentration has a higher burst pressure. Interestingly, even though the polyamide layer typically has a thickness of less than 1 µm, a comparison of Tables 1 and 2 indicates that the deposit of a polyamide layer on top of PES substrates remarkably enhances their burst pressures. The PES-TFC hollow fiber membranes have burst pressures about 10–40% higher than their corresponding substrates.

The surprising increase in burst pressure may result from (1) the enhanced mechanical properties of the inner-layer region due to the interfacial polymerization and (2) different structure failure mechanisms for these two kinds of membranes during burst tests. As aforementioned, during the interfacial polymerization, capillary pressure and a fast-flowing TMC solution flowing stream occur simultaneously. These may render the interfacial polymerization not only on top of the inner surface but also within the pores near the inner-surface region, and thus strengthen the mechanical properties of the inner-layer region and improve the overall mechanical properties of PES-TFC hollow fiber membranes. As a result, the reinforced inner-layer region has a higher burst pressure because it can dissipate the burst stresses more uniformly and effectively than the original PES hollow fiber substrates. In addition, the inner bulk structure of the original PES substrate may deform partly from within due to the combined effects of (1) the weak macrovoid bulk structures and (2) the build-up of the hydraulic pressure within the bulk structure because its outer surface is denser than the inner surface (or the influx water from inner surface is larger than the outflux water from the outer surface). By contrast, the PES-TFC membranes might not have such deformation because the inner polyamide selective layer is much denser than the outer surface of the TFC membrane (or the influx water from the inner surface is much smaller than the outflux water from the outer surface).

### Comparison of burst pressures and benchmark

**Fig. 6:**
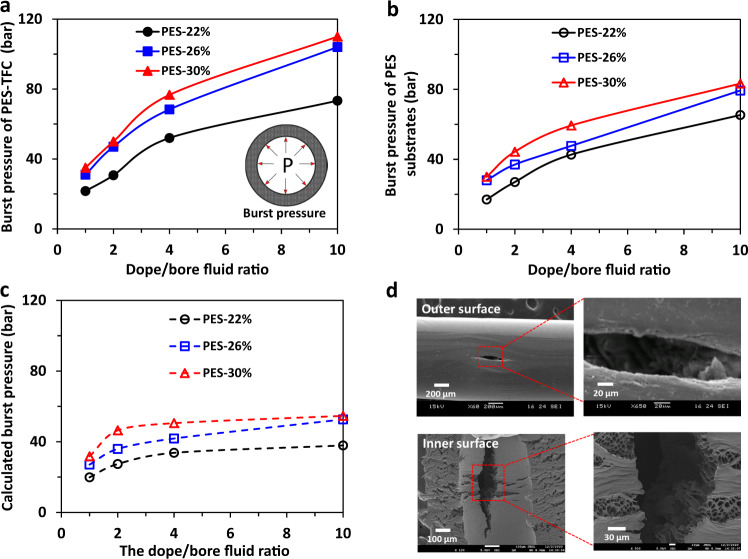
Burst pressures of hollow fiber membranes as a function of the dope to bore fluid ratio and the morphology of the burst membrane. **a** The burst pressures of PES-TFC hollow fiber membranes. **b** The burst pressures of PES hollow fiber substrates. **c** Calculated burst pressure according to Barlow’s equation (Eq. 9) based on the dimension and maximum tensile stress of the substrates. **d** FESEM images of the representative burst PES-TFC hollow fiber (PT22-D).

Figure 6 compares the burst pressures obtained from experiments (Tables 1 and 2) and calculated by Barlow’s equation (Eq. 9). The calculated burst pressures are lower than those obtained experimentally for both PES-TFC membranes and PES substrates. The calculated burst pressures are comparable to the experimental results at low burst pressures (e.g., <50 bar). However, when the dope/bore fluid ratio increases, the asymmetry of the resultant membranes increases. It leads to a great deviation between the calculated and experimental burst pressures as high as 100% at a very high burst pressure (e.g., 110 bar). Since Barlow’s equation is typically applied to estimate the burst pressure of isotropic tubes^29^, one may need to adjust the safety factor of the Barlow’s equation to make a more accurate estimation for the burst pressure of asymmetric hollow fiber membrane. Figure 6d shows the FESEM images of the representative burst PES-TFC hollow fiber (PT22-D). A narrow and long opening can be observed at the outer and inner surfaces of the burst (broken) fiber. The enlarged inner surface shows that the PES substrate is exposed and the polyamide selective layer is displaced due to the outward expansion of the fiber under a high pressure or burst pressure (e.g., ~73 bar).

A benchmark of burst pressures for various membranes is presented in Supplementary Table 3. To the best of our knowledge, the as-developed PES-TFC hollow fiber membranes are the strongest hollow fiber ever reported with the highest burst pressure of up to 110 bar. This value is very comparable to the strongest commercial flat sheet membranes with a burst pressure of 120 bar made of a special module design and spacer^3^. In particular, the newly designed TFC hollow fiber membranes are inner selective hollow fibers, which are favorable for scaling-up and large production.

### Performances of RO and structural parameters

**Fig. 7:**
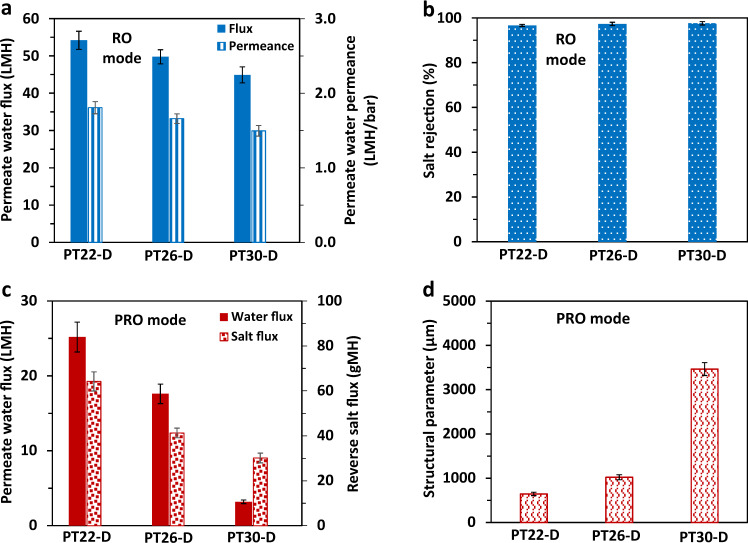
The RO and PRO performances and structural parameters of the optimal PES-TFC hollow fiber membranes. **a**, **b** Permeate water flux, water permeance, and salt (NaCl) rejection of the membranes tested under the RO mode using a 0.3 mol/L NaCl solution as the feed at 30 bar. **c**, **d** Permeate water flux, reverse salt flux, and the structural parameters of the membranes tested under the PRO mode using a 1.2 mol/L NaCl solution as the draw solution at 20 bar. Error bars represent mean absolute errors based on at least three replicate experiments.

The optimal PES-TFC hollow fibers (PT22-D, PT26-D, and PT30-D) were selected to further evaluate their performances for saline dewatering via RO operation mode and investigate their structural parameters (S parameter) by the pressure retarded osmosis (PRO) mode. The results are presented in Fig. 7. As shown in Fig. 7a, b, under the RO mode using a 0.3 mol/L salt solution as the feed at 30 bar, the permeate water permeances of the three hollow fiber membranes are about 1.5 LMH/bar, which is ~50% of their corresponding pure water permeance (PWP) as presented in Table 2. Nevertheless, their salt rejections remain high and are around 97%, which is about 1% less than the rejections evaluated by the standard method using a feed solution of 2000 ppm or 0.034 mol/L (see Table 2). The decreases in permeate water permeance and salt rejection are due to the increase in salt concentration of the RO feed. When using a 0.3 mol/L salt solution as the RO feed, considerable external concentration polarization (ECP) and the internal concentration polarization (ICP) occur, thus reducing the effective driving force across the membrane. Therefore, the water permeances decrease significantly.

The structural parameter of a semi-permeable membrane represents characteristics of the thickness and tortuosity of the membrane support^27,47,48^, and can be estimated by performing the PRO tests^27,48^. As described in Fig. 7c, under the PRO mode using a 1.2 mol/L NaCl solution as the draw solution at the lumen side and DI water as the feed at the shell side, the water permeates to the lumen side while the salt reversely transports from the draw solution in the lumen side to the shell side. The structural parameters of PT22-D, PT26-D, and PT30-D membranes are about 650, 1000, and 3500 μm, respectively. The significant increase in structural parameter, particularly for the PT30-D membrane, is majorly ascribed to the denser PES substrates (P26-D and P30-D) as presented in Fig. 3. The denser substrate has more tortuous diffusion paths, thus leads to have a higher structural parameter. The increases in structural parameters of PT26-D and PT30-D membranes are consistent with the lower PWP values of their substrates (see Fig. 4).

### Performances of OARO

The optimal PES-TFC hollow fiber membranes (PT22-D, PT26-D, and PT30-D) that possess the highest burst pressure at their specific PES concentrations were selected for OARO tests. All TFC membranes were conditioned and stabilized at 30 bar for at least 30 min prior to the OARO measurements. Although they can withstand a pressure of at least 70 bar, 30 bar is the maximum operating pressure used in this study because of the limitations of the pump and the experimental setup.

**Fig. 8:**
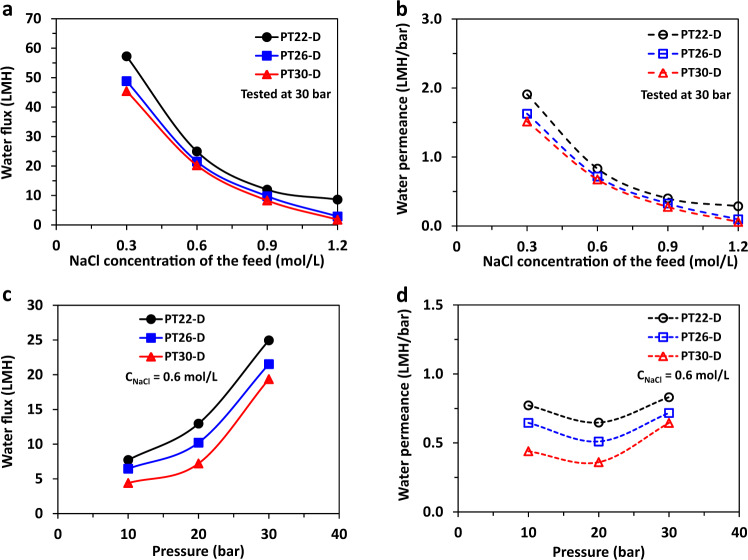
The OARO performances of the optimal PES-TFC hollow fiber membranes. **a**, **b** The effects of salt (NaCl) concentration on water flux and water permeance. The NaCl concentration was the same for the feeds in the lumen and shell sides of hollow fibers, and the operating pressure was 30 bar. **c**, **d** The effects of operating pressure on water flux and water permeance. The feed and sweep streams were 1.2 mol/L NaCl solutions.

The OARO performances of the optimal PES-TFC membranes are shown in Fig. 8. Generally, as described in Fig. 8a, b, both water flux and water permeability decrease dramatically as the NaCl concentration increases. For example, the water flux of the PT22-D membrane declines from 57.2 to 8.6 LMH while the corresponding water permeance declines from 1.91 to 0.29 LMH/bar. The water flux of the PT26-D membrane declines from 48.8 to 2.8 LMH while its water permeance declines from 1.91 to 0.10 LMH/bar. The water flux of the PT30-D membrane decreases from 45.4 to 1.7 LMH while its water permeability decreases from 1.51 to 0.06 LMH/bar. Such decreases in water flux and permeance could be ascribed to two reasons: (1) the increase of osmotic pressure in the feed solution from 14.7 to 58.7 bar as the NaCl concentration rises from 0.3 to 1.2 mol/L; (2) the ICP and ECP reduce the effective driving force across the membrane. On the inner surface (or lumen side) of the TFC hollow fiber, the salts are rejected by the polyamide selective layer and accumulate in its vicinity. Thus, the salt concentration near the inner surface is higher than its bulk concentration in the lumen. Such a phenomenon is known as ECP^49,50^. On the outer surface (or shell side), the water permeates through the selective layer, diluting the salt concentration in the cross-section of the membrane substrate. As a result, the salt concentration behind the polyamide selective layer is lower than its bulk concentration in the sweep solution. Such a phenomenon is known as ICP^50–52^. Both ECP and ICP increase the osmotic pressure difference (Δ*π*) and thus reduce the effective driving force across the membrane under the OARO operation mode. In particular, ICP is believed to be the dominant factor that causes the decrease in water flux and permeance. This is because (1) all ECPs are possibly similar because the flow rates at the shell and lumen sides are kept the same for all cases and (2) ECP declines as the permeate water flux decreases.

As shown in Fig. 8a, b, at a given salt concentration, the water flux and water permeability of the three optimal membranes follow the order of PT22-D > PT26-D > PT30-D. This is because the structural parameters of the three membranes are in the order of PT22-D < PT26-D < PT30-D (see Fig. 7d). Since a larger structural parameter leads to a lower water flux or permeance for a membrane (e.g., evidenced by the PWP values of their substrates in Fig. 4), it may take a longer time for the water permeated from the lumen side to mix with the sweep solution diffused from the shell side. In other words, for the membrane with a larger structural parameter, it may suffer from a severer ICP effect. Therefore, the detrimental ICP effect of the optimal membranes is in the order of PT22-D < PT26-D < PT30-D. The TFC membrane with a smaller structural parameter or made from a weaker substrate with a higher PWP suffers from a less diluting or ICP effect, thereby having a higher effective driving, and thus a higher water flux and water permeance.

The effects of the operating pressure on water flux and water permeance are depicted in Fig. 8c, d. To mimic seawater, a NaCl solution of 0.6 mol/L was chosen to study the effects of operating pressure. The water flux increases from the lowest value of 4.4 LMH at 10 bar for PT30-D to the highest one of 24.9 LMH at 30 bar for PT22-D. The monotonic augment of water flux is due to the increase in operating pressure or driving force. Interestingly, as illustrated in Fig. 8d, the water permeance displays a V-shape trend. The decline-and-increase trend in water permeance may arise from the combined effects of ICP and membrane expansion at high pressure. As the operating pressure increases, the water flux increases, leading to a severer ICP diluting effect. As a result, the water permeability declines. On the other hand, the TFC hollow fiber is subject to a certain degree of expansion because a high pressure (e.g., >20 bar) is applied at the lumen side. It not only radially expands the elastic hollow fiber membrane but also thins the polyamide selective layer, leading to a higher water permeance while being able to maintain the salt rejection^53–55^. Thus, the increase in water permeance overtakes the decrease induced by ICP at 30 bar.

In the OARO mode, the sweep stream can mitigate the osmotic pressure of the feed across the membrane. As a result, the osmotic pressure difference (Δ*π*) is reduced. Therefore, it is clear that water can be recovered from saline water even though the operating pressure is lower than the osmotic pressure of salt water by applying the OARO strategy. The water flux can be further increased by increasing the operating pressure because the as-developed TFC hollow fiber membranes can withstand a hydraulic pressure of much more than 30 bar. It is worth noticing that a higher applied hydraulic pressure (e.g., > 30 bar) is needed when the solution/feed is more concentrated or a higher water recovery is required. Although the PT30-D membrane has the highest burst pressure, its structure parameter is much higher than the PT22-D and PT26-D ones. In real applications, one may choose the PT26-D membrane for the OARO process because it has a decent burst pressure but a relatively low structure parameter.

In conclusion, a series of strong TFC hollow fiber membranes have been developed in this study for saline dewatering via reverse osmosis (RO) and osmotically assisted reverse osmosis (OARO) processes. By adopting the strategies of tuning the hollow fiber dimension and adjusting the host polymer (PES) concentration, an ultra-strong hollow fiber membrane with a burst pressure of about 110 bar is demonstrated. Besides the dope composition and membrane spinning conditions, dope rheology such as die swell and chain relaxation also play important roles in determining phase inversion mechanism, membrane morphology, and mechanical properties. The increase in mechanic strength of the hollow fibers does come with the price of the increase in structural parameter or internal concentration polarization (ICP). Nevertheless, this work may pave a new venue for developing ultra-strong polymeric TFC hollow fiber membranes for RO, OARO, and other applications.

## Methods

### Fabrication of PES-TFC hollow fiber membranes

The materials used in this work can be found in the Supplementary Information. The PES hollow fiber substrates were prepared by adopting the dry-jet wet-spinning process^19,27^. The polymer dope consisting of PES, NMP, PEG400, DI water (H_2_O), and CaCl_2_ were prepared according to the dope compositions presented in Supplementary Table 1. Briefly, the PES polymer was added into a mixture of NMP and PEG400, then stirred overnight under a temperature of 60 to ~80 °C. Subsequently, the mixture of CaCl_2_ and water was added dropwise to the pre-dissolved polymer solution and continuously stirred until the solution became homogeneous. The as-dissolved polymer dope was filled into a syringe pump (ISCO) and degassed overnight. The hollow fiber spinning was carried out according to the conditions and parameters shown in Supplementary Table 1.

The as-spun PES hollow fiber substrates were immersed in tap water for 3 days with a daily water change to remove the residual solvent and additives. Then the fibers were soaked in a glycerol aqueous solution (glycerol/DI water = 50/50 wt%) for at least 48 h as the post treatment. Subsequently, the treated hollow fiber substrates were hung and dried in air at ambient temperature for at least 2 days. The fibers without the glycerol treatment were freeze-dried and used for characterizations.

The lab-scale membrane modules, each containing 3 hollow fiber membranes with an effective length of about 15 cm, were prepared. The inner-selective TFC hollow fiber membranes were fabricated by performing the interfacial polymerization (IP) at the lumen side of the fibers. Prior to the interfacial polymerization, the membrane modules were soaked in DI water for at least 1 h to pre-wet the substrates. The procedures for fabricating the PES-TFC hollow fibers were as follows: (1) An aqueous precursor solution consisting of 2 wt% MPD and 0.1 wt% SDS was circulated through the lumen side of substrates for 3 min, followed by air purge for 5 min to remove the excessive precursor solution. (2) A hexane solution containing 0.15 wt% TMC was circulated through the lumen side of the substrates for 5 min, whereby the TMC contacted and reacted with MPD that had been absorbed in the inner surface of the substrates. The resultant thin polyamide selective layer was formed on the inner surface of the hollow fiber membrane. (3) The as-formed PES-TFC hollow fibers were dried by air purging for 1 min to remove the residual hexane. The dried PES-TFC hollow fiber membranes were kept in DI water for characterizations and RO, PRO and OARO tests.

### Measurements of PWP, salt rejection, and permeability

The pure water permeance (PWP), in L/(m^2^ h bar) or LMH/bar, of the PES hollow fiber substrates and PES-TFC membranes were measured by circulating the DI water at the lumen side of the fibers, while the water permeate was collected from the shell side of the fibers. The PWP designated as *A* can be calculated from the following equation,

1 $$A=\frac{\varDelta V}{M\varDelta t\varDelta P}$$

where, Δ*V* is the volumetric change of the permeate within a testing time interval (Δ*t*), *M* is the effective membrane area that allows the water to permeate through, Δ*P* is the pressure difference across the hollow fiber membrane.

The salt (NaCl) rejection of the PES-TFC membrane was determined by applying a 2000 ppm NaCl solution as the feed at the lumen side. The conductivities of the feed and permeate were monitored using a conductivity meter (SCHOTT Instruments, Lab 960). Then the conductivities were converted into corresponding salt concentrations. Thus, the salt rejection (*R*) can be estimated by the following equation:

2 $$R=\left(1-\frac{{C}_{{\rm{p}}}}{{C}_{{\rm{f}}}}\right)\times 100 \%$$

where, *C*_p_ and *C*_f_ are the salt concentrations of the permeate and the feed, respectively.

The salt permeability (*B*) in L/(m^2^ h) or LMH was determined according to the equation below^29,56^:

3 $$B=\frac{1-R}{R}(\varDelta P-\varDelta \pi )A$$

where, Δ*π* is the osmotic pressure difference across the membrane.

The PWP of the substrate was measured at a transmembrane pressure of 1 bar using a conventional ultrafiltration (UF) setup. The PWP and salt rejection of the PES-TFC membranes were evaluated by employing a RO setup as described elsewhere^29,56^. To avoid the break of membranes, PES-TFC membranes fabricated from substrates with condition A (e.g., the dope/bore fluid flow rate = 1) were conditioned and tested at low pressures in the range of 10–20 bar. Unless stated otherwise, the PWP of all other PES-TFC membranes were conditioned at 30 bar for about half an hour and measured at 20 bar and about 25 °C.

### RO and PRO tests

The optimal hollow fiber membranes (PT(20-30)-D) were selected to further characterize their performances and properties under RO and PRO operation modes. The detailed experimental conditions for RO and PRO modes were tabulated in Supplementary Table 2. The structural parameter (*S* parameter) was evaluated by conducting the PRO tests^27,48^.

The water permeation flux, *J*_w_ (MLH), was measured according to the following equation:

4 $${J}_{{\mathrm{w}}}=\frac{\varDelta {V}_{f}}{M\varDelta t}$$

where, the Δ*V*_*f*_ is the volumetric change of the sweep/feed solution.

The reverse salt flux can be determined according to the following equation:

5 $${J}_{{\rm{s}}}=\frac{\varDelta ({V}_{{\rm{f}}}{C}_{{\rm{f}}})}{M\varDelta t}$$

where, *V*_f_ and *C*_f_ are the volume and salt concentration of the solution, respectively.

The structural parameter (*S*) of the membrane is related to the thickness (*t*) of the membrane support (e.g., the wall thickness of the hollow fiber substrate), the tortuosity (*τ*), and bulk porosity (*ε*) of the membrane support. These parameters have the relationship as follows:

6 $$S=\frac{t\tau }{\varepsilon }$$

However, in practice, the accurate tortuosity (*τ*) of the membrane support is impossible to measure. Therefore, the structural parameter was estimated indirectly by the following equation:

7 $${J}_{{\rm{w}}}=A\left(\frac{{\pi }_{{\rm{d}}}-{\pi }_{{\rm{f}}}\exp \big(\frac{{J}_{{\rm{w}}}S}{D}\big)}{1+\frac{B}{{J}_{{\rm{w}}}}\big[\exp \big(\frac{{J}_{{\rm{w}}}S}{D}\big)-1\big]}-\varDelta P\right)$$

where, *π*_d_ and *π*_f_ are the osmotic pressures of the draw solution and the feed solution, respectively; *D* is the solute diffusion coefficient (e.g., for NaCl, *D* = 1.61 × 10^−9^ m^2^/s). The structural parameter (*S*) can be obtained by solving the Eq. 7.

### OARO tests

The OARO tests were performed by circulating salt solutions with the same concentration at both the lumen and shell sides of the TFC hollow fiber membranes under a counter flow mode. The external hydraulic pressure was applied at the lumen side, while the shell side was maintained at atmospheric pressure. Supplementary Table 2 summarizes the detailed experimental conditions for OARO tests.

### Membrane characterizations

The bulk viscosities of the PES spinning dopes were determined at room temperature (25 °C) via a rotary viscometer (Lamy Rheology Instrument, Black One; range: 20 to 156,000,000 mPa s or cP). The bulk porosity of the hollow fiber substrate was calculated according to the following equation:

8 $$\varepsilon =\left(1-\frac{4m}{\pi L{\rho }_{P}(O{D}^{2}-I{D}^{2})}\right)\times 100 \%$$

where *ε* (%) represents the bulk porosity; *L*, *OD*, and *ID* are the length, the outer and inner diameters of the hollow fiber membrane, respectively. *ρ*_*P*_ is the density of PES (1.37 g/cm^3^) determined according to the Archimedes’ principle^57^, and *m* is the mass of the hollow fiber sample. This equation (Eq. 8) is applicable for the freeze-dried pristine hollow fiber substrate, which was not treated with the glycerol aqueous solution, to minimize the effects of trace amounts of the residual solvent and additives on porosity.

The burst pressures of the PES hollow fiber substrates and PES-TFC hollow fiber membranes were determined using a manual hydro pressure test pump or hand pump (KYOWA, Japan; Model: T300NDX, range: 0–300 bar). Briefly, as shown in Supplementary Fig. 3, the hollow fiber membrane module was connected to the hose of the hand pump. Firstly, the effluent valve 2 was opened so that water was pumped into the lumen side of the fibers to eliminate air. Then the valve 2 was closed, and water was pumped slowly into the hollow fibers. The pressure within the lumen side of the fibers was built up as the driving force was increased by pushing the handle downward with hands. When the gauge pressure suddenly decreased, indicating the burst pressure was reached. Usually, a bursting sound was heard.

The burst pressure of the pipe structure can also be approximately estimated according to Barlow’s equation^29,30^, as shown below:

9 $$P=\frac{2xT}{OD\times {F}_{{\rm{S}}}}$$

where, *P* represents the burst pressure, *x* represents the wall thickness, *T* is the maximum tensile stress and *F*_s_ is the safety factor (typically, *F*_s_ = 1). Other membrane characterizations such as field emission scanning electron microscope (FESEM), physical and mechanical properties were presented in the Supplementary Information. Unless stated otherwise, average testing results of at least three membranes or membrane modules were reported in this work, and the average errors or deviations of the data are less than 10%.

## Supplementary information

Supplementary Information

## Data Availability

The authors declare that all data supporting the findings of this study are available within this paper and its Supplementary Information file.
