# Supplementary material for: Ultra-strong polymeric hollow fiber membranes for saline dewatering and desalination
Source: Nat Commun. 2021 Apr 20;12:2338. doi: 10.1038/s41467-021-22684-1 (PMC8058345; doi:10.1038/s41467-021-22684-1)
Supplement: Supplementary file 1 — Supplementary Information [file 41467_2021_22684_MOESM1_ESM.pdf]

Supplementary Information

Ultra-strong polymeric hollow fiber membranes for saline dewatering and desalination

Liang et al.

Supplementary methods

Materials

The polyethersulfone (PES, 3100P) was bought from Solvay. Prior to being used for studies, it was dried under vacuum at 80 °C for 24 h to remove moisture. N-methyl pyrrolidone (NMP) (EMPLURA®, ≥99.5%), sodium chloride (NaCl, 99.5%), calcium chloride ($CaCl_2$) were acquired from Merck. Polyethylene glycol 400 (PEG 400, M_w = 400 g/mol) was purchased from Sigma-Aldrich. Glycerol (industrial grade) was supplied by Aik Moh Paints & Chemicals (Singapore). Trimesoyl chloride (TMC, >98%) and phenylenediamine (MPD, >99%) were bought from Tokyo Chemical Industry. Sodium dodecyl sulphate (SDS, >97%) was purchased from Fluka. Hexane (> 99.9%) was ordered from Fisher Chemicals. Unless stated otherwise, the chemicals were used as received. Deionized (DI) water was generated by a water purification system (Millipore, Elix).

Other membrane characterizations: FESEM, physical and mechanical properties

The morphologies of the membranes were investigated by a field emission scanning electron microscope (FESEM, JEOL, JSM-6700LV). Each membrane sample was immersed in liquid nitrogen for a certain time. Then the frozen membrane was fractured and/or slashed using a razor blaze. The fractured sample was coated using a platinum (Pt) sputter coater (JEOL JFC-1300) before the FESEM observation. The mechanical properties of the PES hollow fiber substrates were measured using an Instron universal testing system (Model 3342, Instron), while their outer and inner diameters were determined by a stereo microscope (OLMPUS, Model: SZX2-ILLT).

Supplementary Table 1. Conditions and parameters for the fabrication of hollow fiber membrane substrates.

Air gap	5.0 (cm)											
External coagulant	Tap water											
Temperature	Room temperature (25 °C)											
Take-up speed	Free fall (~ 3 m/min)											
Bore fluid	DI water											
PES concentration of dope solution (wt%)	22				26				30			
Dope flow rate (ml/min)	2.0	2.0	2.0	2.0	2.0	2.0	2.0	2.0	2.0	2.0	2.0	2.0
Bore fluid flow rate (ml/min)	2.0	1.0	0.5	0.2	2.0	1.0	0.5	0.2	2.0	1.0	0.5	0.2
Ratio of dope to bore fluid flow rate	1	2	4	10	1	2	4	10	1	2	4	10
Code name of hollow fiber	P22-A	P22-B	P22-C	P22-D	P26-A	P26-B	P26-C	P26-D	P30-A	P30-B	P30-C	P30-D
	PES-22%				PES-26%				PES-30%			

Supplementary Table 2. Experimental conditions for reverse osmosis (RO), pressure-retarded osmosis (PRO) and osmotically assisted reverse osmosis (OARO) tests.

Testing mode	Lumen side		Shell side	
RO	Feed solution	0.3 mol/L, NaCl	Sweep solution	DI water
	Flow rate (ml/min)	200	Flow rate (ml/min)	200
	Pressure (bar)	30	Pressure (bar)	0
	Temperature (°C)	25	Temperature (°C)	25
PRO	Draw/feed solution	1.2 mol/L, NaCl	Sweep/feed solution	DI water
	Flow rate (ml/min)	200	Flow rate (ml/min)	200
	Pressure (bar)	20	Pressure (bar)	0
	Temperature (°C)	25	Temperature (°C)	25
OARO	Feed solution	0.3-1.2 mol/L, NaCl	Sweep solution	0.3-1.2 mol/L, NaCl
	Flow rate (ml/min)	200	Flow rate (ml/min)	200
	Pressure (bar)	10-30	Pressure (bar)	0
	Temperature (°C)	25	Temperature (°C)	25

Supplementary Table 3. A comparison of the burst pressure for different membranes.*

Configuration of membrane	Burst pressure (bar)	Pressure type	Application	Reference
Flat sheet (commercial)	43-120	Press	RO/OARO	1
Flat sheet (commercial)	85	Press	RO	2
Flat sheet (commercial)	48	Press	FO/NF	3
Hollow fiber (commercial)	69	Out to in	RO	4
Hollow fiber (commercial)	29	Out to in	PRO	4
Hollow fiber	7.5	Out to in	FO/PRO	5
Hollow fiber	95	Out to in	OSRO	6
Hollow fiber	24	In to out	PRO	7
Hollow fiber	30	Out to in	PRO	8
Hollow fiber	35	In to out	PRO	9
Hollow fiber	73-110	In to out	OARO	This work

* Reverse osmosis (RO), osmotically assisted reverse osmosis (OARO), forward osmosis (FO), nanofiltration (NF), pressure retarded osmosis (PRO), organic solvent reverse osmosis (OSRO).

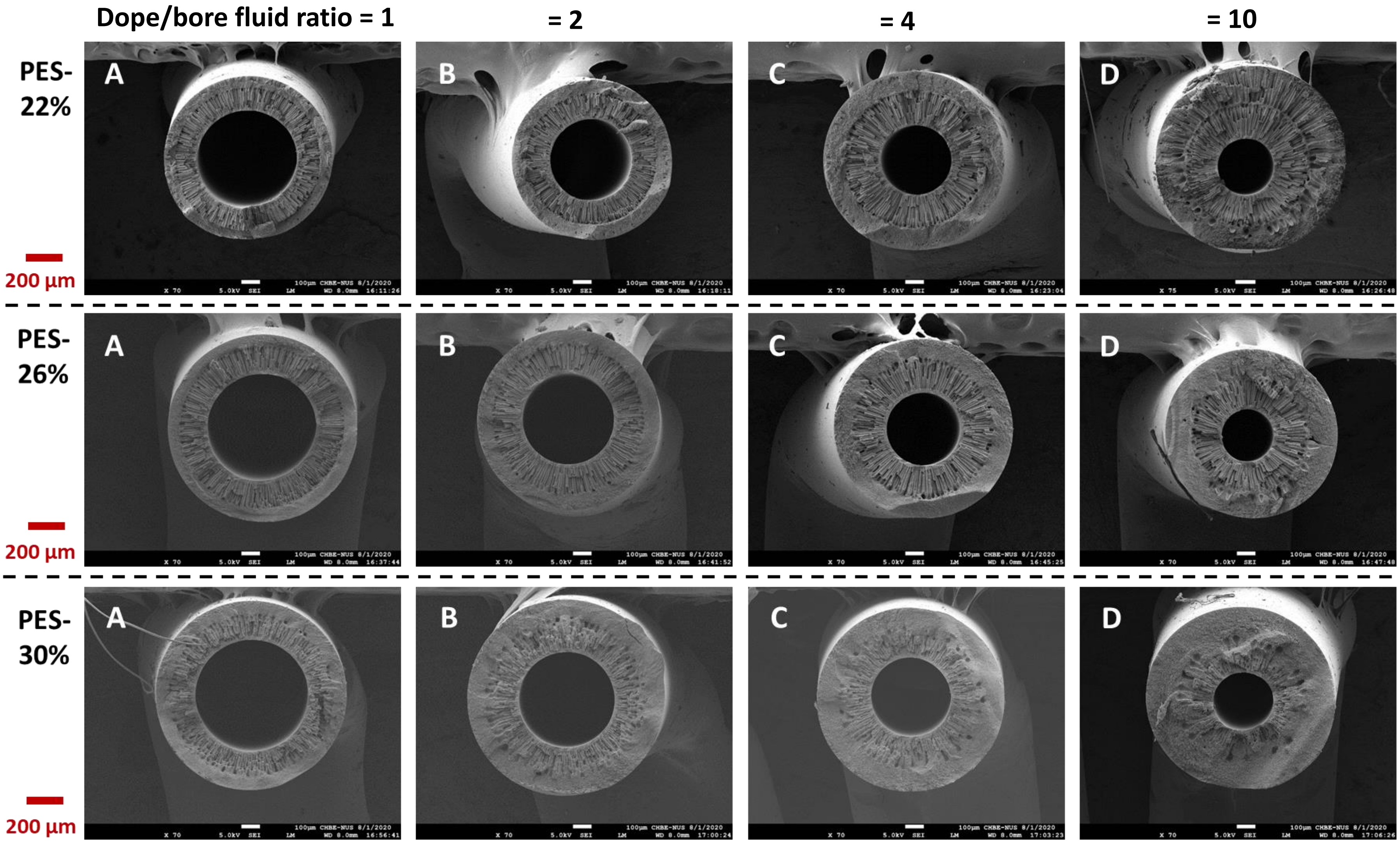

Supplementary Figure 1. FESEM morphologies of cross-sections of the as-spun PES hollow fiber membrane substrates

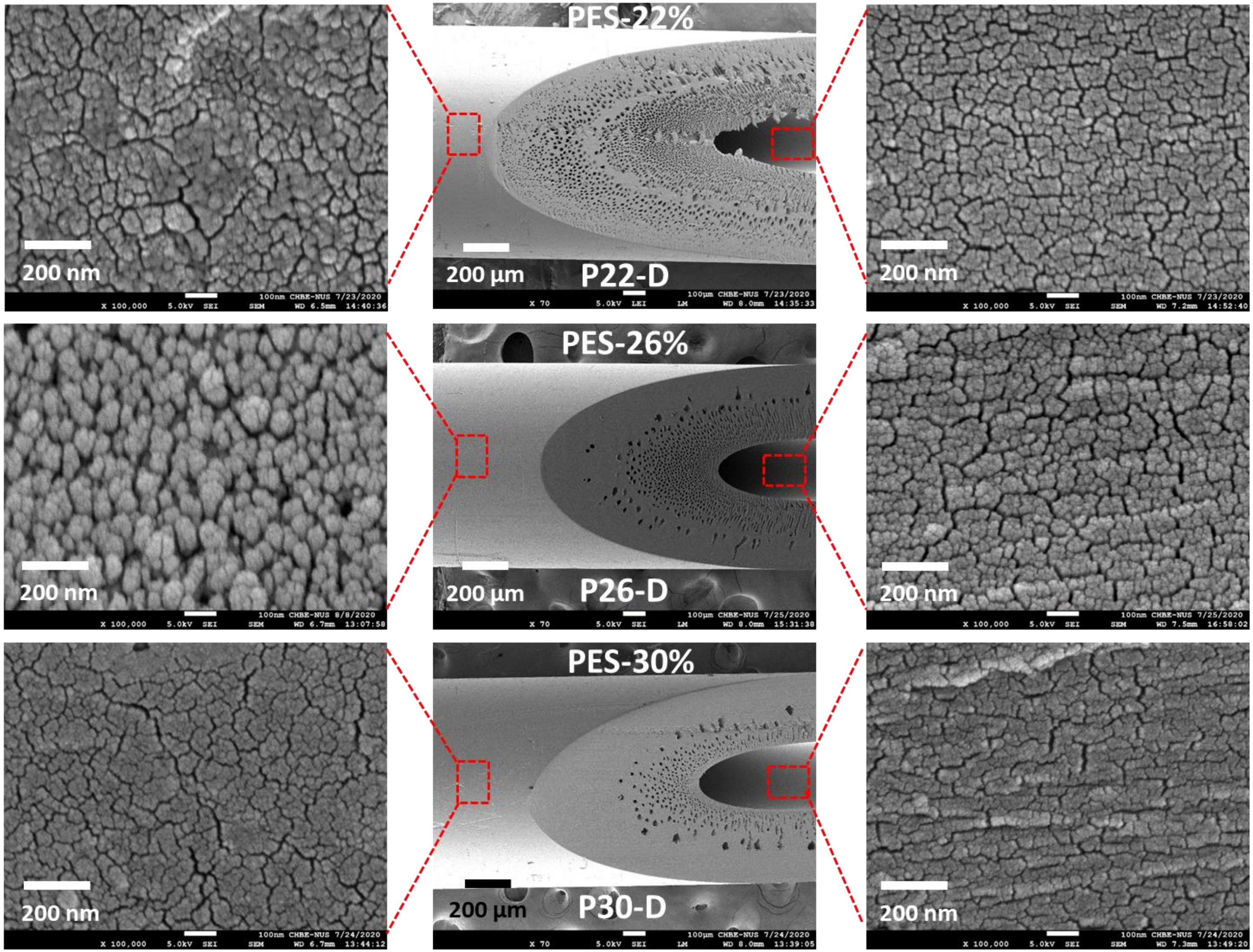

Supplementary Figure 2. FESEM morphologies of the outer surface and inner surface of representative PES hollow fiber substrates. The hollow fibers were spun at the dope to bore fluid ratio of 10.

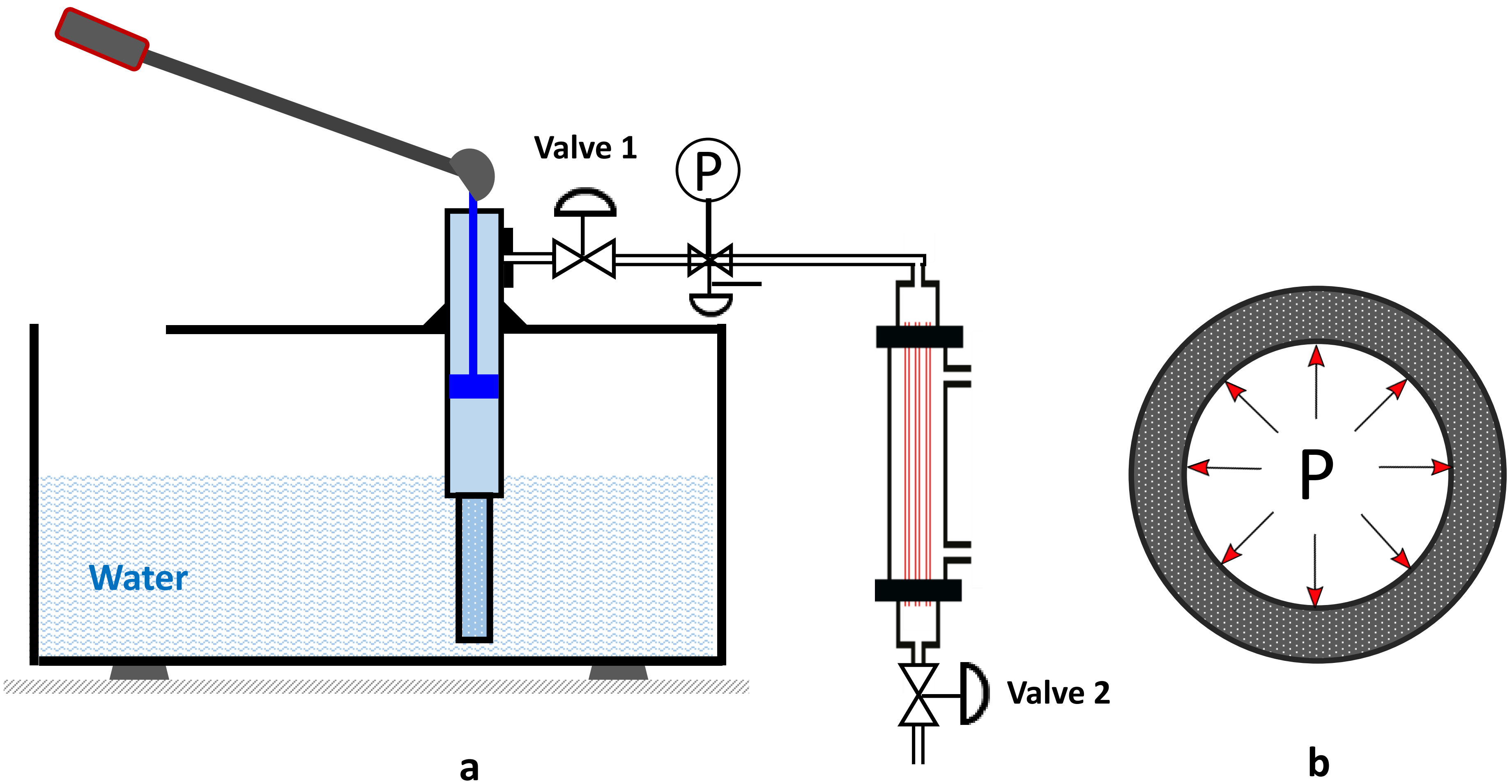

Supplementary Figure 3. The illustration of the setup for burst pressure tests. **a** The schematic diagram of the hydraulic hand pump to test the burst pressure of hollow fiber membranes. **b** The schematic illustration of the pressure (in to out) within the lumen side of the hollow fiber.

Supplementary References

1. Peters CD, Hankins NP. Osmotically assisted reverse osmosis (OARO): Five approaches to dewatering saline brines using pressure-driven membrane processes. *Desalination* **458**, 1-13 (2019).

2. Chen X, Yip NY. Unlocking high-salinity desalination with cascading osmotically mediated reverse osmosis: energy and operating pressure analysis. *Environ. Sci. Technol.* **52**, 2242-2250 (2018).

3. Kim J, Kim DI, Hong S. Analysis of an osmotically-enhanced dewatering process for the treatment of highly saline (waste)waters. *J. Membr. Sci.* **548**, 685-693 (2018).

4. Kumano A, Marui K, Terashima Y. Hollow fiber type PRO module and its characteristics. *Desalination* **389**, 149-154 (2016).

5. Fu FJ, Zhang S, Sun SP, Wang KY, Chung TS. POSS-containing delamination-free dual-layer hollow fiber membranes for forward osmosis and osmotic power generation. *J. Membr. Sci.* **443**, 144-155 (2013).

6. Jang HY, Johnson JR, Ma Y, Mathias R, Bhandari DA, Lively RP. Torlon® hollow fiber membranes for organic solvent reverse osmosis separation of complex aromatic hydrocarbon mixtures. *AlChE J.* **65**, (2019).

7. Li X, Chung TS. Thin-film composite P84 co-polyimide hollow fiber membranes for osmotic power generation. *Appl. Energy* **114**, 600-610 (2014).

8. Cheng ZL, Li X, Liu YD, Chung T-S. Robust outer-selective thin-film composite polyethersulfone hollow fiber membranes with low reverse salt flux for renewable salinity-gradient energy generation. *J. Membr. Sci.* **506**, 119-129 (2016).

9. Wan CF, Yang T, Gai W, Lee YD, Chung TS. Thin-film composite hollow fiber membrane with inorganic salt additives for high mechanical strength and high power density for pressure-retarded osmosis. *J. Membr. Sci.* **555**, 388-397 (2018).
